# Bridging The Gap: Essential Role of Parents and Caregivers In Their Child's Management in the Pediatric Intensive Care Unit

**DOI:** 10.7759/cureus.66177

**Published:** 2024-08-05

**Authors:** Manojkumar G Patil, Sampada Tambolkar, Neha Tyagi, Shradha Salunkhe, Shailaja V Mane

**Affiliations:** 1 Pediatrics, Dr. D. Y. Patil Medical College, Hospital, and Research Centre, Dr. D.Y. Patil Vidyapeeth (Deemed to be University), Pune, IND

**Keywords:** parent education, parent attitude, picu, patient and family-centered care, family-centered care

## Abstract

The importance of parental involvement in the care and management decisions made for children hospitalized in the pediatric intensive care unit (PICU) is examined in this editorial. Initial days and weeks in a PICU can be challenging for the child and family due to the emotional intensity and medical complexity of the therapy a child receives. Regardless of the result, families may feel uncertain and anxious that their child may die or have a terrible outcome. The majority of pediatric patient deaths in hospitals happen in the PICU. Recognizing and supporting the crucial role of parents or caretakers in informed decision-making and management of their child’s condition is essential for advancing prevention, detection, and treatment efforts.

## Editorial

Numerous research studies have found the possibility that some parents may develop symptoms of anxiety, sadness, or post-traumatic stress disorder when their child is admitted to the pediatric intensive care unit (PICU). It is recommended that parents have follow-up appointments with PICU health specialists following their child's release to promote their psychological well-being. There may be long-term psychological repercussions and a link between parents' psychological anguish and their children's long-term psychological well-being. A collaborative approach to healthcare, family-centered care (FCC), upholds the parental role and the parents' engagement in their child's upbringing. As part of its mission to facilitate "more effective, efficient, and empathic pediatric health care," the Family and Children's Center encourages dialogue between families and pediatric healthcare providers [1]. The following five elements are recognized: 1) information sharing; 2) hearing from parents; 3) decision-making foror with parents; 4) role-negotiating; and 5) personalizing communication. These themes draw attention to several differences in how parents view and desire to be involved in their child's care by professionals [2]. From parents as caregivers to parents as recipients of care, parental preferences for engagement vary in the areas of information sharing, decision-making, and power sharing. The PICU culture is gradually changing to involve parents in all facets of their child's care and to promote collaborations between parents and members of the healthcare team as a result of the drive for family-centered pediatric care.

Even though the concept of FCC is regarded as the gold standard of care in pediatrics, its application faces many difficulties, particularly in the PICU context. First, it is challenging to assess the efficacy of the FCC because there is disagreement over its definition and since definitions frequently enumerate a wide range of concepts [3]. Second, it can be challenging to predict definitive outcomes for patients in the PICU due to their unstable and/or unpredictable medical conditions. This can cause parents to become more anxious and create more communication difficulties [4]. Further study is needed to ensure that the care provided in the PICU to patients and their families is family-centered and encompasses all fundamental principles. Parents of children in the PICU have expressed feelings of discontent with the exchange of information and communication, mostly due to the uncertainty around their child's prognosis and the therefore frequently evolving treatment plan. Physicians must acknowledge that parents experience stress due to the unpredictable nature of a child's PICU trajectory. Additional measures are required to enhance parent satisfaction with information sharing and promote consistent communication. Furthermore, parents of kids who spent a lot of time in the PICU have expressed that they would rather have the same nurses care for their kids every time [5]. It is necessary to conduct research to elucidate the environmental and cultural obstacles that have impeded the implementation of consistent nurse-caregiver assignments in the PICU.

To conclude, healthcare providers should keep in mind that parents anticipate that they will be able to participate in their child's care, that their requests for dependable nurses will be honored, and that they will regularly have open, sincere, and caring information exchanged through family meetings and rounds. As stated by the Institute for Patient and Family-Centered Care, providing high-quality, patient- and family-centered intensive care requires considering the physical and cultural environment of the PICU [6].

## References

[1] Hutchfield K (1999). Family-centred care: a concept analysis. J Adv Nurs.

[2] Kuo DZ, Houtrow AJ, Arango P, Kuhlthau KA, Simmons JM, Neff JM (2012). Family-centered care: current applications and future directions in pediatric health care. Matern Child Health J.

[3] Mikkelsen G, Frederiksen K (2011). Family-centred care of children in hospital - a concept analysis. J Adv Nurs.

[4] Johnson BH (2000). Family-centered care: facing the new millennium. Interview by Elizabeth Ahmann. Pediatr Nurs.

[5] Baird J, Rehm RS, Hinds PS, Baggott C, Davies B (2016). Do you know my child? Continuity of nursing care in the pediatric intensive care unit. Nurs Res.

[6] Committee on Hospital Care and Institute for Patient- and Family-Centered Care (2012). Patient- and family-centered care and the pediatrician's role. Pediatrics.

